# Roles of omental and bone marrow adipocytes in tumor biology

**DOI:** 10.1080/21623945.2019.1643189

**Published:** 2019-07-23

**Authors:** Yoon Jin Cha, Ja Seung Koo

**Affiliations:** Department of Pathology, Yonsei University College of Medicine, Seoul, South Korea

**Keywords:** Bone marrow, omentum, adipokines, metastasis, lipids, tumours

## Abstract

Accumulating evidence highlights the importance of interactions between tumour cells and stromal cells for tumour initiation, progression, and metastasis. In tumours that contain adipocyte in their stroma, adipocytes contribute to modification of tumour microenvironment and affect metabolism of tumour and tumour progression by production of cytokines and adipokines from the lipids. The omentum and bone marrow (BM) are highly adipocyte-rich and are also common metastatic and primary tumour developmental sites. Omental adipocytes exhibit metabolic cross-talk, immune modulation, and angiogenesis. BM adipocytes secrete adipokines, and participate in solid tumour metastasis through regulation of the CCL2/CCR2 axis and metabolic interactions. BM adipocytes also contribute to the progression of hematopoietic neoplasms. Here, we here provide an overview of research progress on the cross-talks between omental/BM adipocytes and tumour cells, which may be pivotal modulators of tumour biology, thus highlighting novel therapeutic targets.

**Abbreviations:** MCP-1, monocyte chemoattractant protein 1IL, interleukinSTAT3, signal transducer and activator of transcription 3FABP4, fatty acid binding protein 4PI3K/AKT, phosphoinositide 3-kinase/protein kinase BPPAR, peroxisome proliferator-activated receptorPUFA, polyunsaturated fatty acidTAM, tumour-associated macrophagesVEGF, vascular endothelial growth factorVEGFR, vascular endothelial growth factor receptorBM, bone marrowBMA, bone marrow adipocytesrBMA, regulated BMAcBMA, constitutive BMAUCP-1, uncoupling protein-1TNF-α, tumour necrosis factor-alphaRANKL, receptor activator of nuclear factor kappa-Β ligandVCAM-1, vascular cell adhesion molecule 1JAK2, Janus kinase 2CXCL (C–X–C motif) ligandPGE2, prostaglandin E2COX-2, cyclooxygenase-2CCL2, C-C motif chemokine ligand 2NF-κB, nuclear factor-kappa BMM, multiple myelomaALL, acute lymphoblastic leukemiaAML, acute myeloid leukemiaGDF15, growth differentiation factor 15AMPK, AMP-activated protein kinaseMAPK, mitogen-activated protein kinaseAPL, acute promyelocytic leukemiaCCR2, C-C motif chemokine receptor 2SDF-1α, stromal cell-derived factor-1 alphaFFA, free fatty acidsLPrA, leptin peptide receptor antagonistMCD, malonyl-CoA decarboxylase.

## Introduction

The tumour microenvironment (TME) affects tumour biology through various biological processes. Adipocytes are a particularly important component of the TME exerting both systemic and local effects on tumour growth and progression when tumours contain adipocyte in their stroma [1,2]. Among the human organs containing adipocytes, the omentum and bone marrow (BM) show particularly high enrichment of adipocytes. Importantly, the omentum and BM are also frequent metastatic sites of tumours as well as common sites of primary tumour development. Adipocytes of the omentum and BM have different origins compared to those derived from other sites, and thus exhibit specialized functions that affect tumour biology. Therefore, it is essential to identify the characteristics of omental and BM adipocytes, and their impacts on tumour biology. We here review the research progress on these adipocyte types with a focus on their roles in metastasis through metabolic interactions, and cross-talk with immune and tumour cells. Gaining a greater understanding of the underlying molecular and cellular mechanisms can highlight novel potential therapeutic targets for tumour treatment.

## Basic characteristics of the omentum

The omentum is a visceral adipose tissue, mostly composed white adipose tissue that consisted of vascularized connective tissue, and doubled mesothelial layered membranous and translucent tissue[3]. The omentum, a main lipid storage and a source of bioactive factors, involves in the immune response and fluid exchange [4–6]. Milky spots are the primary functional units of the omentum [7,8], which are distributed along with the blood vessel networks[9]; detailed information of milky spots has been reviewed previously[10]. The structural components of milky spots are fibroblasts, adipocytes, mesothelial cells, endothelial cells, macrophages, stromal cells, and high endothelium of the vein, whereas the migratory components include lymphocytes, granulocytes, and monocytes[11]. Milky spots have recently come into the research spotlight as the main implantation sites of omental cancer cell metastasis. Since adipocytes composing the milky spots are also distributed around the milky spots, various interactions could occur between adipocytes and milky spots that might mediate metastatic processes.

Omental adipocytes have distinct characteristics from adipocytes derived from other sites. In particular, the lipolysis action of catecholamine is increased in omental adipocytes compared with subcutaneous adipocytes, whereas the anti-lipolytic action of insulin and prostaglandin is more prominent in subcutaneous adipocytes than in omental adipocytes[12]. Expression of prostaglandin synthesis- or signalling-related genes is also higher in omental adipocytes than in subcutaneous adipocytes[13]. Similarly, transcriptomics studies revealed that the expression of adipogenesis and lipid metabolism-related genes differs between omental and subcutaneous adipose tissues [14–16], and proteomics analysis revealed differential expression of proteins related to lipid metabolism, oxidation-reduction, and lipid transport between the tissues[17]. Lipidomic analysis of obese individuals showed that compared to subcutaneous adipose tissue, omental adipose tissue contained 54% and 34% more cholesterol and cholesterol epoxide[18]. Moreover, omental and subcutaneous adipocytes have different origins, and only the former express Wilms’ tumour gene (WT-1)[19]; indeed, the presence of a WT-1-positive mesothelial cell layer in the omentum is considered to be the possible origin of these adipocytes. Omental adipocytes differ from other visceral adipocytes in variable circumstances. Omental adipocytes of obesity patients are larger in size, and have lower capillary density compared with periaortic adipocytes[20]. Expression of IL-18, HGF, and MIF is higher in omental adipocytes than in periaortic adipocytes[20]. Omental preadipocytes are also different from mesenteric preadipocytes in having lower replicative potential, lower differentiation, lower adipogenic transcription factor expression and higher TNF-α induced apoptosis[21].

## Roles of omental adipocytes in tumour development and metastasis

**Figure 1.**
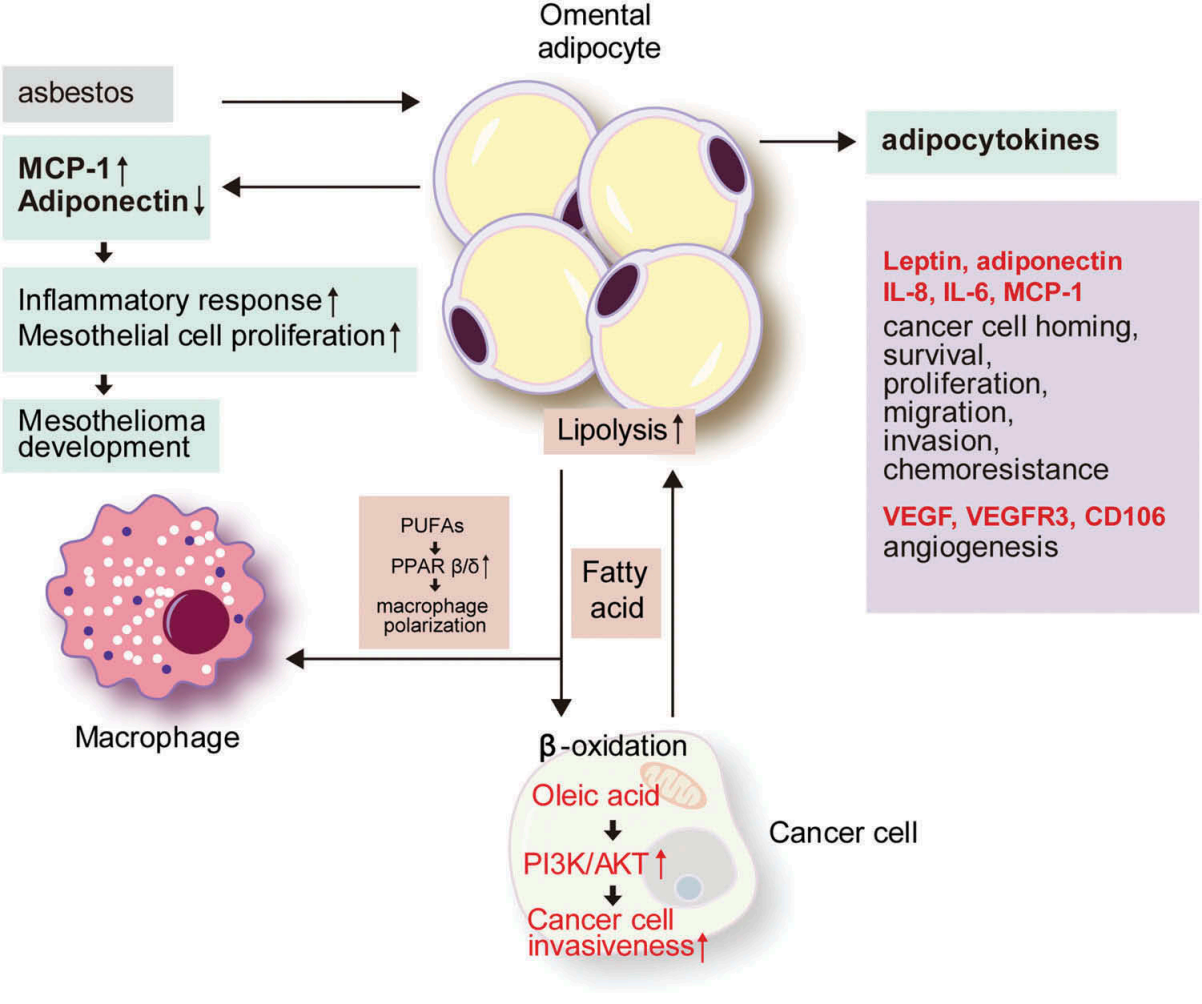
The role of omental adipocytes in tumour development and metastasis. In omental adipocytes exposed to asbestos, MCP-1 secretion is increased and adiponectin secretion is reduced, resulting in an inflammatory reaction and mesothelial cell hyperplasia that induces the development of mesothelioma. Among adipokines from omental adipocytes, leptin, adiponectin, IL-8, IL-6, and MCP-1 induce tumour cell homing, survival, proliferation, migration, invasion, and chemo-resistance in metastatic tumour cells in the omentum. VEGF, VEGFR3, and CD106 enhance angiogenesis. Metastatic tumour cells in the omentum promote lipolysis, and the generated fatty acids are transferred to tumour cells and used in β-oxidation. Oleic acid activates the PI3K/AKT pathway and promotes cancer cell invasiveness. PUFAs activate PPARβ/δ in macrophages and polarizes them into tumour-associated macrophages. MCP-1, monocyte chemoattractant protein 1; IL, interleukin; VEGF, vascular endothelial growth factor; VEGFR, vascular endothelial growth factor receptor; PUFA, polyunsaturated fatty acid.

Most of the tumours found in the omentum are metastatic tumours, and primary omental tumours are extremely rare. Malignant mesothelioma is a representative example of an omental primary tumour. It is suggested that adipocytes can play a key role in tumour promotion during asbestos-induced mesothelial carcinogenesis. Upon exposure to asbestos, the levels of proinflammatory cytokines such as monocyte chemoattractant protein 1 (MCP-1) increase, while anti-inflammatory cytokines such as adiponectin decrease in the adipocytes, resulting in an inflammatory environment. Since MCP-1 promotes mesothelial cell proliferation, these inflammatory stimuli could trigger a carcinogenesis process under a proliferative state[22] (Figure 1).

The most common carcinomas that metastasize to the omentum include ovarian cancer, colorectal cancer, gastric cancer, and pancreatic cancer[23]. In particular, 80% of ovarian serous carcinomas exhibit omental metastasis[24]. The main route of omental metastasis is via direct intraperitoneal seeding rather than hematogenous dissemination. Hence, interactions between metastatic tumour cells and the environment of the implantation site are key factors in promoting omental metastasis. To establish omental metastasis, cancer cells seeding in the omentum must pass through several checkpoints, including survival in the peritoneal cavity, evasion of the immune system, reattachment at the secondary site, and angiogenesis[23]. Accumulating evidence has demonstrated that omental adipocytes influence every step of the omental metastasis process.

### Adipokines

Adipocyte-derived metabolites and bioactive peptides are collectively referred to as adipokines, with more than 600 identified to date[25]. In general, adipokines play roles in the regulation of appetite, fat distribution, insulin secretion, energy expenditure, inflammation, and blood pressure[26]. In the adipose tissue, adipokines participate in adipogenesis, immune cell migration, and adipocyte metabolism [27,28]. In tumours, adipokines act via adipokine receptors on tumour cells[29]. In an ovarian cancer mouse model, omental adipocytes promote tumour cell homing to omentum after intraperitoneal tumour cell injection through the actions of interleukin (IL)-8, IL-6, MCP-1, and adiponectin secreted by omental adipocytes[30]. The adipokines secreted from omental adipocytes activate pro-survival pathway, p38, and signal transducer and activator of transcription 3 (STAT3) in ovarian cancer cells. Omental adipocytes show increased IL-8 secretion, which promotes the invasiveness of ovarian cancer cells [30,31]. Upon establishment of the omental metastasis of ovarian cancer, secreted IL-8 and TP53 upregulate the expression of fatty acid binding protein 4 (FABP4), which enhances the fatty acid uptake of tumour cells to promote cancer cell growth[31]. Moreover, the peritumoral adipokine profile changes of omental metastases of pancreatic cancer have been described; specifically, leptin expression increases to enhance metastasis[32]. Secretory factors from omental adipocytes promote the reprogramming of pancreatic cancer cells, such as an increase of extracellular matrix and adhesion molecules, resulting in the promotion of cancer cell growth, migration, invasion, and chemo-resistance[33].

### Lipid supply and metabolic interactions

Omental adipocytes supply lipids to tumour cells and also support their survival and proliferation in the omentum. An *in vitro* study with ovarian cancer cells demonstrated the lipid transfer from adipocytes to tumour cells, which was enhanced for omental adipocytes compared with subcutaneous and mesenteric adipocytes[30]. Lipids entering tumour cells supply the energy required for tumour cell proliferation by β-oxidation[30]. To meet the high energy demand, tumour cells upregulate the lipolysis of adipocytes, and their secretion of free fatty acids and glycerol[30]. FABP4 [31] and CD36 [34] are the main lipid transporters during the interaction of tumour cells and adipocytes. After lipid transfer, the size of the adipocytes decreases by consuming lipid droplets. Thus, omental adipocytes become smaller during omental metastasis, and ultimately disappear to become replaced by the metastatic tumour cells, referred to as the ‘omental cake.’[35] In gastric cancer, oleic acids are transferred to tumour cells from omental adipocytes. Intracellular oleic acid activates the phosphoinositide 3-kinase/protein kinase B (PI3K/AKT) pathway of tumour cells, which promotes their invasiveness[36]. The metabolites produced as by-products during lipid metabolism also affect tumour cell metabolism, including glycerol, a by-product of lipolysis, which can act as a substrate for the glycolytic pathway to promote metastatic tumour cell growth and adaptation [37,38].

### Cross-talk with immune cells

As mentioned above, a milky spot, the functional unit of the omentum, is composed of diverse cell types, including immune cells such as macrophages, mast cells, and B- and T-lymphocytes, that are important in the immune response of the omentum [39,40]. The milky spot is the initial tumour cell attachment site for metastasis[9], and is thus critical for tumour growth and survival. Milky spots are only found in the omentum and splenoportal adipose tissue among the peritoneal adipose tissues [35,41]. Interactions between adipocytes and peritoneal macrophages have been shown to contribute to ovarian cancer cell metastasis. Peroxisome proliferator-activated receptor (PPAR)β/δ is involved in cancer-associated processes and activated by various lipid ligands[42]. Analyzing of tumour-associated macrophages driven from ovarian cancer ascites showed constantly upregulated PPARβ/δ with impaired ligand response[43]. Ovarian cancer ascites was rich in polyunsaturated fatty acids (PUFAs), particularly linoleic acid[43], which could be derived from omental adipocytes. PUFAs could serve as ligand of PPARs, and PUFA/PPARδ structure promoted their FA sensing ability[44]. PPARδ promotes lipid accumulation in macrophages[45], and this may explain the high concentration of PUFAs and constant upregulation of PPARβ/δ in tumour-associated macrophages (TAMs) in ovarian cancer ascites[43], which play a pro-tumorigenic role in tumour microenvironment. This kind of fatty acid accumulated in TAM is now recognized as tumour promotor [46,47].

### Angiogenesis

Milky spots exist along with the vascular network of the omentum and at sites of active angiogenesis. In general, angiogenesis in milky spots occurs by vascular endothelial growth factor A (VEGFA) secreted from omental mesothelial cells and macrophages. In a hypoxic condition, the omental adipocytes secrete VEGF, vascular endothelial growth factor receptor (VEGFR) 3, and CD105 [9,48,49] to induce angiogenesis and thereby promote cancer survival and chemoresistance [50,51]. Microarray analysis with the Oncomine assay revealed higher expression levels of *VEGFR1, VEGFR2, CD31*, and *CD34* in omental metastatic cancer tissues than in the primary ovarian cancer[52].

### BM adipocytes (BMA)

BMA are an important component of the BM with diverse roles, and the proportion of BMA in the BM fluctuates under various conditions. The number of adipocytes tends to increase with age, obesity, malnutrition, and stimulation of drugs or radiation [53–56]. Approximately 70% of the adult BM volume is occupied by BMA[57], which can be classified into inducible or regulated BMA (rBMA) and constitutive BMA (cBMA) that differ in terms of development, lipid saturation, gene expression, and vascular density [58,59]. rBMA are characterized by their proximal location and red marrow, whereas cBMA are characterized by their more distal location and yellow marrow. BMA originate from BM mesenchymal stem cells that are bi-potent progenitors, with the ability to differentiate into adipocytes and/or osteoblasts[60]. BMA show several typical adipocyte phenotype characteristics. The brown-like phenotype BMA provide energy to hematopoietic and mesenchymal components [61] and express uncoupling protein-1 (UCP-1)[62], whereas white-like phenotype BMA play roles in the storage and process of triglycerides, and regulate fatty acid metabolism [61,63,64]. This phenotypic difference has been suggested to be based on the location, with BMA in the long bone and vertebrae corresponding to the white and brown phenotype, respectively[65]. BMA secrete various adipokines, including hormones, cytokines, and fatty acids, that affect bone remodelling, energy regulation, and insulin metabolism [66,67]. The secretory profile of BMA also differs from that of adipocytes from other sites: BMA show lower expression of adiponectin mRNA compared with that of extramedullary adipocytes [68,69] but have higher expression levels of tumour necrosis factor-alpha (TNF-α) and IL-6 than those in visceral adipocytes[68], accompanied by higher pro-angiogenic and pro-apoptotic profiles[70]. In bone remodelling, BMA secrete leptin, adiponectin, and chemerin; leptin and adiponectin induce the osteoblastic differentiation and proliferation of mesenchymal stem cells, while chemerin suppresses osteogenesis[65]. Moreover, the abundant saturated fatty acids in BMA induce osteoblast dysfunction and apoptosis, and osteoclastogenesis is facilitated by TNF-α and receptor activator of nuclear factor kappa-Β ligand (RANKL) secreted from BMA[65].

## Roles of BMA in solid tumour metastasis

**Figure 2.**
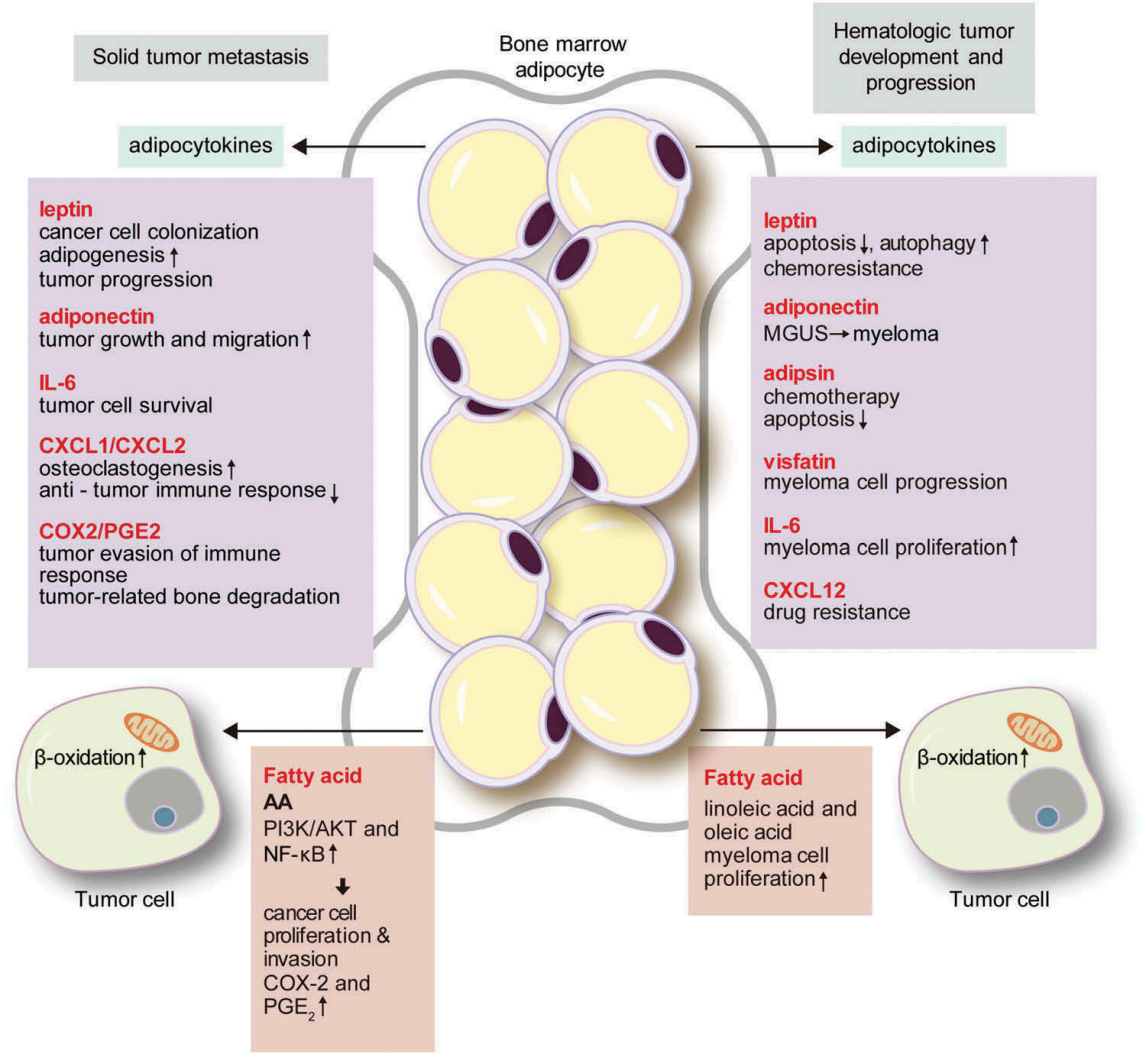
The role of bone marrow adipocytes in solid tumour metastasis and hematologic tumour development. Bone marrow adipocytes are involved in solid tumour metastasis via the secretion of various adipokines. Leptin enhances cancer cell colonization and adipogenesis, which induces tumour progression. Adiponectin promotes tumour growth and migration. IL-6 increases tumour cell survival. The CXCL1/CXCL2 axis increases osteoclastogenesis, and suppresses the anti-tumour immune response. The COX-2/PGE2 axis represses the immune response, and induces tumour-related bone degradation. Adipocytokines are also involved in hematologic tumour development and progression. Leptin suppresses apoptosis and activates autophagy, which induces chemo-resistance. Reduction of adiponectin secretion promotes the progression of MGUS to myeloma. Adipsin and CXCL12 participate in drug resistance. IL-6 and visfatin are involved in myeloma cell progression. Tumour cells of solid tumours and hematologic tumours receive fatty acids transferred from the adipocytes. In solid tumours, AA activates the PI3K/AKT and NF-κB pathways that induce cancer cell proliferation and invasion. AA also activates COX-2 and PGE2 that repress the immune response and induce tumour-related bone degradation. In hematologic tumours, linoleic acid and oleic acid increase the proliferation of myeloma cells. CXCL, (C–X–C motif) ligand; COX-2, cyclooxygenase-2; PGE2, prostaglandin E2; MGUS, monoclonal gammopathy of undetermined significance; IL, interleukin; PI3K/AKT, phosphoinositide 3-kinase/protein kinase B; NF-κB, nuclear factor-kappa B.

The bone is one of the most common metastatic sites of cancers, especially for prostate, breast, and lung cancers[71]. The incidence of bone metastasis at autopsy is 75–80% for prostate cancer [71,72], and 65–75% for breast cancer[73]. In prostate cancer, old age and obesity are the primary risk factors of metastasis [74–76], which are factors that increase the numbers of BMA. The main metastatic sites of prostate cancer are the axial skeleton and long bone metaphysis, which is a site of active bone remodelling with high marrow cellularity[77]. Thus, a metabolically active BM with abundant adipocytes may be the preferred site of metastasis. Previous studies indicated that metastatic cells forming colonies in the bone were attracted by an adipocyte-rich and metabolically active red BM [78,79]. Thus, substantial research has focused on the contribution of the BMA in the bone metastasis of solid tumours, revealing various mechanisms. Here, we focus on the secretion of adipocytokines and lipid transfer as the representative mechanisms (Figure 2).

### Adipocytokines secreted by BMA

BMA secrete various adipocytokines such as leptin, adiponectin, IL-1β, IL-6, vascular cell adhesion molecule 1 (VCAM-1), TNF-α, and VEGF, [80] which influence cancer cell biology. Leptin indirectly affects prostate cancer cell growth via promoting bone resorption, [81,82] and increased expression of IL-1β drives cancer cell colonization of BMA in breast cancer[83]. Leptin is also important for BMA generation. Leptin binds to the leptin receptor on BM stem cells, and activates the Janus kinase 2 (JAK2)/STAT3 pathway to trigger adipogenesis [84,85]. Leptin secreted into BMA binds leptin receptor on the tumour cells, which induces tumour progression [86–88]. Indeed, tumour patients show greater amounts of adiponectin secreted by BMA[55]. Although adiponectin generally acts as a tumour suppressor[89], some studies showed that adiponectin enhanced tumour growth and migration [90,91]. This dual effect may be derived from the difference of adiponectin receptor isoforms[90]. BMA secrete abundant IL-6[92], which induces the epithelial-mesenchymal transition in tumour cells via the JAK2/STAT3 pathway[93], and strengthens the metastatic potential of tumour cells via PI3K/AKT[94]. Peculiarly, IL-6 can be activated by an extracellular soluble form of IL-6 receptor (IL-6R), without a receptor of tumour cells[95]. Therefore, if the soluble form IL-6R along with IL-6 are secreted by BMA, they would have a strong effect on the metastatic process. (C–X–C motif) ligand (CXCL)1 and CXCL2, chemokines produced by BMA, enhance osteoclastogenesis in metastatic prostate cancer and prolong tumour cell survival[96]. Upregulated expression of IL-6 in malignant melanoma cells increased osteoclastogenesis, which could induce the proliferation of tumour cells[97]. CXCL1 and CXCL2 also participate in immune modulation, acting as chemoattractants for macrophages, neutrophils, and CD11b+Gr1+ cells [98,99]. These immune cells have CXCL2 receptors and suppress the anti-tumour immune response[100]. Furthermore, the cyclooxygenase-2 (COX-2)/prostaglandin E2 (PGE2) signalling axis induces inflammation and immune suppression, facilitating tumour evasion of the host immune response[101]. Overexpression of COX-2 and PGE2 is the main cause of tumour-related bone degradation in bone metastasis [102,103]. In a breast cancer mouse model, an increase of the COX-2 level increased tumour colonization and osteoclastogenesis, and induced lytic bone metastasis[104]. Adipokines also participate in angiogenesis. When prostate cancer cells were exposed to BMA, VEGF expression increased[105], and CCL2 secreted by adipocytes was found to promote breast cancer progression by inducing angiogenesis[106].

### Lipid transfer and characteristic lipid components

BMA provide the lipid source required for the proliferation, migration, and invasion of solid cancer cells [83,105]. In a cell line study, prostate cancer cells co-cultured with BMA were found to be surrounded by lipid droplets and showed increased expression levels of the lipid transfer-associated molecules FABP4, CD36, and perilipin 2[105]. Microarray analysis with Oncomine data also revealed increased expression levels of *FABP4* and *CD36* in metastatic prostate cancer compared to those of the primary cancer[52]. In addition, CD36 expression was increased in breast cancer and prostate cancer cells co-cultured with BMA[105]. An *in vivo* study also supported the lipid transfer from BMA to tumour cells: in the early phase of bone metastasis, the number of BMA increased by adipogenesis, but the number of BMA with abundant lipid droplets decreased during tumour progression[107]. Bone metastasis is more frequent in the rBMA-enriched region than in the cBMA-enriched region. rBMA can respond flexibly during metabolic interactions with tumour cells, as they readily adapt to their environment. BMA also influence the metabolic phenotype of metastatic prostate cancer cells. Previous study on prostate cancer showed that BMA induced Warburg-type metabolism in cancer cells in paracrine manner, along with decreased mitochondrial oxidative phosphorylation[108]. Glycolytic enzymes, *ENO2, LDHa, PDK1, HK2*, and GLUT1 were upregulated in prostate cancer cells that were co-cultured with adipocytes[108]. Exposure of prostate cancer cells to BMA induced hypoxia-inducible factor 1-alpha signalling and persisted Warburg-type metabolism[108].

The lipid droplets of BMA are composed of large-sized saturated and unsaturated fatty acids, particularly oleic, palmitic, and omega-6 PUFAs, and AA[109]. Some of the fatty acids derived from BMA could impact bone metastasis. For example, AA transferred from BMA to prostate cancer cells activated the PI3K/AKT and nuclear factor-kappa B (NF-κB) signalling pathways, and promoted cancer cell proliferation and infiltration [110,111]. Moreover, AA is also related to the expression of COX-2 and PGE2, thus playing a role in the COX-2/PGE2 signalling axis [110,112].

## Roles of BMA in hematologic neoplasms

The studies reviewed above clearly demonstrate the roles of interactions between solid tumours and BMA in bone metastasis. However, hematologic neoplasms such as multiple myeloma (MM) and leukaemia are primarily derived from the BM, which could be the primary niche of these neoplasms. In solid tumours, elevated leptin level is associated with cancer risk[113]. Also, in hematologic neoplasms, such as MM, leptin was revealed to have pro-tumour effect[80]. Moreover, previous study showed that adipocytes protected acute lymphoblastic leukaemia (ALL) cells from vincristine, a chemotherapeutic agent, by sequestering lipophilic vincristine, as well as upregulating anti-apoptotic proteins, Pim-2 and Bcl-2[114]. In MM patients, myeloma cells induced adipogenesis from osteoblast progenitor cells, and increased the number of BMA, which contributed to MM progression[115]. In acute myeloid leukaemia (AML) patients, BM mesenchymal stem cells tended to differentiate into adipocytes[116], which implies that tumour microenvironment favours adipocyte-rich state. So far, BMA have been considered as negative regulators in BM microenvironment and hematopoiesis[117]. Preferred differentiation to adipocytes of BM mesenchymal stem cells may lead to the depletion of hematopoietic stem cell niche, and also facilitate tumour growth. Size reduction of BMA surrounding AML cell line is caused by lipolysis of adipocytes by leukemic cells, which leads to increase of free fatty acid utilized by leukemic cells. Growth differentiation factor 15 (GDF15) level, secreted from AML cells, induces morphological remodelling of BMA [118] and lipolytic pathway to generate fatty acid for tumour proliferation[119] (Figure 2).

### Adipokines

An epidemiologic study showed that low adiponectin and high leptin levels are associated with an increased tumour risk in multiple myeloma[113]. *In vitro*, myeloma cells co-cultured with adipocytes showed enhanced proliferation and migration, and leptin was found to clearly play a role in this process[120]. Leptin activates the AKT/STAT3 pathway, increases Bcl-2 levels, and suppresses caspase-3 and in turn apoptosis, which collectively contribute to the development of chemo-resistance in myeloma[121]. Autophagy, which is also activated by leptin, inhibits chemotherapy-induced apoptosis[122]. Adiponectin activates the AMP-activated protein kinase (AMPK) and mitogen-activated protein kinase (MAPK) pathways in myeloma cells, and reduces the rate of tumour cell proliferation while promoting apoptosis[123]. Decreased adiponectin levels promote the progression to myeloma from the pre-myeloma stage, because a low adiponectin level is not sufficient to properly inhibit acetyl-CoA-carboxylase, a key enzyme of lipid synthesis in tumour cells[124]. Adipsin secreted from adipocytes inhibits chemotherapy-induced apoptosis in myeloma cells by increasing autophagy[108]. Vistafin, a visceral fat-derived protein, has been shown to be related to multiple myeloma progression[125]. IL-6 promotes myeloma cell proliferation both *in vitro* and *in vivo*[126], and the IL-6 level is correlated with myeloma progression[127]. TNF-α independently promoted the proliferation of myeloma cells[128], and induced the expression of CCL2 in myeloma cells together with IL-6[129]. CCL2 leads to the macrophage recruitment that supports myeloma cell survival, drug resistance, and angiogenesis[130].

Cytokines and chemokines secreted by BMA induce proliferation of AML cells. Leptin increases survival of leukemic cells [131,132], and induces proliferation of AML cell lines and blasts [133,134]. In acute promyelocytic leukaemia (APL), leptin from adipocytes suppresses APL cell apoptosis via STAT3 and MAPK pathway[135]. In ALL, stromal cell-derived factor-1 alpha (SDF-1α) secreted by adipocytes binds to CXCR receptor, which induces cytoskeletal remodelling and makes leukemic cells migrate to adipose tissue[136]. Finally, CXCL2 secreted from adipocytes was shown to induce drug resistance in a leukaemia mouse model[137]. These above-mentioned adipokines are released by all different deposits of adipose tissue and are found in the circulation.

### Lipid metabolism and lipid metabolites

Blast cells of AML induce the phosphorylation of hormone-sensitive lipase in BMA, and activate lipolysis, resulting in the increased production of fatty acids. The fatty acids are transferred to AML blasts via FABP4, which help to promote tumour cell proliferation[138]. BMA also promotes fatty acid β-oxidation as well as the expression of the *PPARɣ, FABP4, CD36*, and *BCL2* genes, which collectively inhibit the apoptosis of acute monocytic leukaemia cells[139]. Thus, adipocytes serve as energy source of AML have reduced size, and small BMA size is known as poor prognostic factor in AML[140]. Furthermore, BMA in AML transfer free fatty acids (FFA) to hematopoietic stem cells that make survival and growth of AML blasts[141]. When ALL cell line was co-cultured with adipocytes, FFA produced from adipocyte lipolysis was used by ALL cell, for tumour cell proliferation[142]. Fatty acids have various effects on myeloma: LA and oleic acid induce the proliferation of myeloma cells [143,144], whereas unsaturated fatty acids such as alpha-LA and eicosapentaenoic acid caused myeloma cell death *in vitro*[145], and PUFAs induced the apoptosis of human leukemic cells[146].

## Therapeutic targets of omental and BM adipocytes for tumour treatment

**Figure 3.**
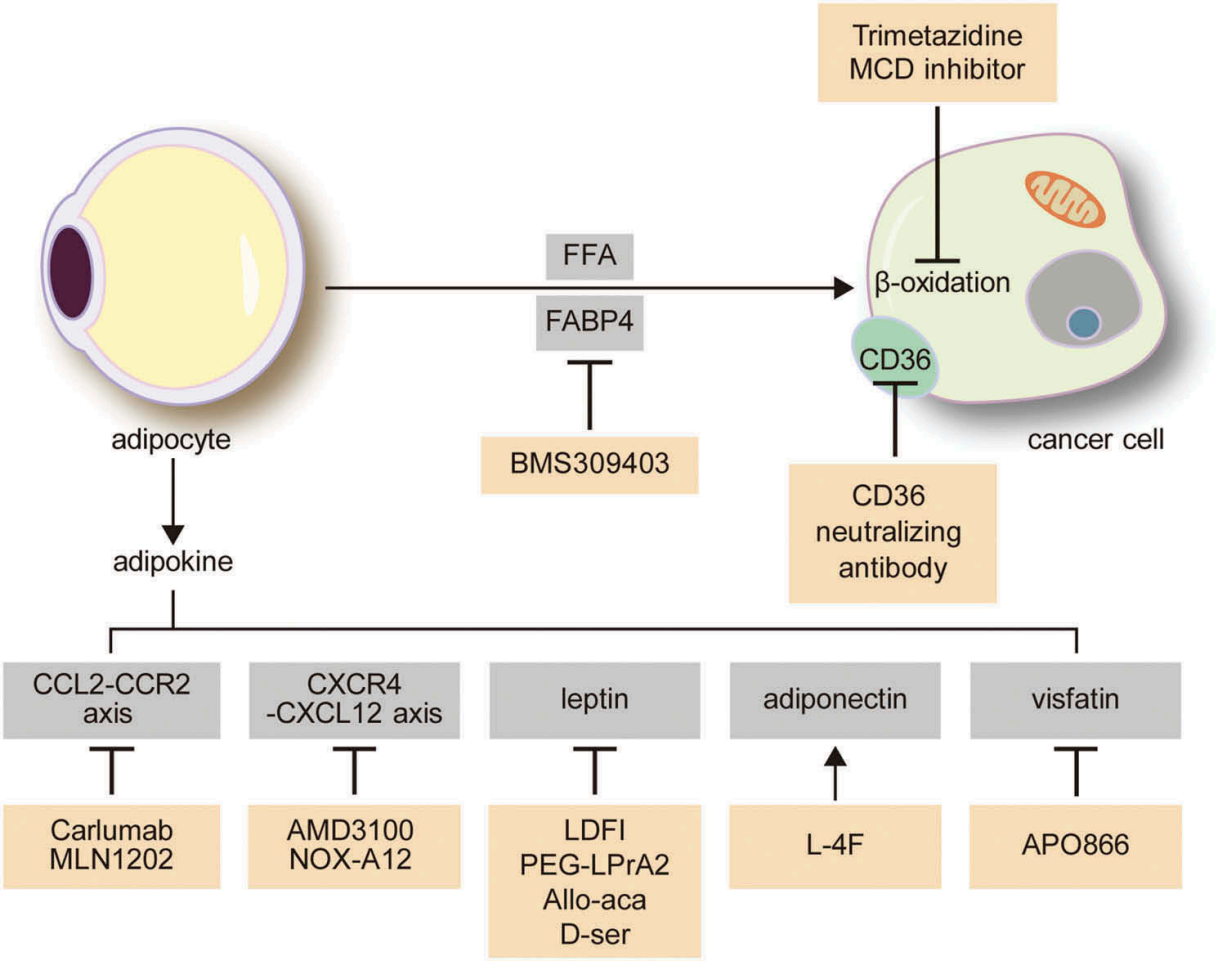
Possible treatment targets for the interaction between cancer cells and adipocytes in the omentum and bone marrow. CCL2/CCR2 axis inhibitors are modulators of adipokines. Calruman and MLN1202 are monoclonal antibodies against CCL2 and CCR2, respectively. CXCR4/CXCL12 axis inhibitors include AMD3100 and NOX-A12, which are inhibitors for CXCR4 and CXCL12, respectively. L-4F, an apolipoprotein mimetic, increases the adiponectin level and has an anti-tumour effect. APO866 is a visfatin inhibitor. Lipid metabolic interactions between tumour cells and adipocytes are potential therapeutic targets. Trimetazidine and malonyl-CoA decarboxylase (MCD) inhibitors are inhibitors of fatty acid β-oxidation in tumour cells. BMS 309403 inhibits FABP4, a fatty acid transporter, and CD36 blocking antibody blocks CD36, a transmembrane protein for fatty acid uptake. CCL2, C-C motif chemokine ligand 2; CCR2, C-C motif chemokine receptor 2; CXCL, (C–X–C motif) ligand; CXCR, (C–X–C motif) receptor; FABP4, fatty acid binding protein 4.

Given the evident roles of omental and BM adipocytes in the various pathways of tumour biology, targeting the interaction between tumour cells and adipocytes could be an effective new cancer treatment strategy (Figure 3).

### Adipokine modulators

Adipokine modulators have shown promising tumour-suppressive effects. Leptin has pro-tumorigenic effects as well as induces in chemoresistance via NF-κB and TGF-β signalling pathways [147,148]. Leptin antagonists include leptin mutant proteins[149], leptin peptide antagonist[149], leptin peptide receptor antagonist (LPrA) [150,151], and Allo-aca and D-ser [152,153]. Among these, leptin peptide antagonist, LDFI, inhibited leptin-induced proliferation of breast cancer cells *in vivo* and *in vitro*[154]. LPrA2 prevented breast cancer in mouse model, associated with reduction of levels of leptin-induced molecules[155]. Allo-aca and its analogue peptide D-ser inhibited leptin-induced proliferation of cancer cells in *in vitro*: breast cancer cell line, MDA-MB231[152], leptin-receptor positive breast and colon cancer cells[153]. AMD3100, a CXCR4 inhibitor, was found to increase the sensitivity to therapy in multiple myeloma cells[156]. NOX-A12, a CXCL12 inhibitor, also increased the sensitivity of chronic lymphocytic leukaemia cells to chemotherapy[157]. Carlumab, monoclonal antibody to CCL2 inhibits CCL2 binding to the CCR2 receptor[158]. Also known as CNTO888, carlumab showed promising antitumor effect in pre-clinical study[159]. Although carlumab was well tolerated in solid tumour patients with lesser adverse effect, unlikely to in vitro study, carlumab expected to have lesser binding affinity in human further study and review are required [160,161]. L-4F, an apolipoprotein mimetic, increased the adiponectin level and displayed a chemotherapeutic effect on myeloma[123], breast cancer[162], and ovarian cancer[163]. APO866, a visfatin inhibitor, induced the apoptosis of myeloma cells, and repressed the rate of tumour cell proliferation[125].

### Lipid metabolism inhibitors

Given the metabolic interactions between adipocytes and tumour cells, targeting of metabolic pathways has been explored as a treatment target. Fatty acids released during lipolysis are transferred to tumour cells and used for energy production via mitochondrial β-oxidation, which enhances tumour progression. Hence, fatty acid oxidation in cancer cells is a promising therapeutic target. A malonyl-CoA decarboxylase (MCD) inhibitor inhibits fatty acid oxidation by increasing the malonyl-CoA level, which is a key inhibitory enzyme of fatty acid uptake in mitochondria, and in turn could reduce the proliferation of human breast cancer cells[164]. In addition, the transportation of fatty acids from adipocytes to tumour cells has been explored as a potential treatment target. BMS 309403, an inhibitor of FABP4, decreased cancer cell proliferation[30], and a CD36 blocking antibody that blocks the acid uptake of CD36 decreased breast cancer cell metastasis and ovarian cancer cell growth [34,165].

## Conclusion and prospects

The omentum and BM are highly enriched in adipocytes and are the main sites of metastasis for various types of solid tumours. The BM is also a primary site of hematologic tumour development. Adipocytes are a component of the TME that dictates tumour development, survival, and progression; thus, targeting adipocytes can be an important strategy for suppressing tumour development, cancer cell survival, and progression. Adipocytes of omentum and BM differ in their origin and location, but both serve as endocrine organs secreting adipokines and are involved in tumour biology. Adipocytes of the omentum and BM secrete various adipokines with pro-tumour effects on growth signalling, angiogenesis, and immune modulation. Furthermore, they transfer lipids to adjacent tumour cells to influence tumour metabolism, and enhance tumour proliferation and survival. Thus, targeting the interaction between tumour cells and adipocytes of the omentum and BM could be an effective tumour treatment. In this regard, IL-6, TNF-α, CXCL12, and CCL2 are therapeutically targetable adipokines, while FABP4 and CD36 are potential targets regarding the metabolic interaction. Further study is required to uncover the detailed relationships between adipocytes of the omentum and BM and their influence on tumour biology, and to identify and validate new potential treatment targets.
